# Perilipin‐1 autoantibodies are a robust marker of acquired lipodystrophy and may precede clinical detection

**DOI:** 10.1111/pai.70026

**Published:** 2025-01-09

**Authors:** Clément Triaille, Fernando Corvillo, Ann Mertens, Ilse Hoffman, Tania Roskams, Margarita López‐Trascasa, Lien De Somer, Kristina Casteels

**Affiliations:** ^1^ Division of Pediatric Rheumatology, Department of Pediatrics University Hospitals Leuven Leuven Belgium; ^2^ Pôle de Pathologies Rhumatismales Systémiques et Inflammatoires, Institut de Recherche Expérimentale et Clinique Université Catholique de Louvain Brussels Belgium; ^3^ Complement Research Group, Hospital La Paz Institute for Health Research (IdiPAZ) La Paz University Hospital Madrid Spain; ^4^ Clinical and Experimental Endocrinology, Department of Chronic Diseases and Metabolism KU Leuven Leuven Belgium; ^5^ Department of Endocrinology University Hospitals Leuven Leuven Belgium; ^6^ Department of Paediatric Gastroenterology & Hepatology & Nutrition University Hospitals Leuven Leuven Belgium; ^7^ Department of Imaging and Pathology KU Leuven Leuven Belgium; ^8^ Departamento de Medicina Universidad Autónoma de Madrid Madrid Spain; ^9^ Department of Pediatrics University Hospitals Leuven Leuven Belgium; ^10^ Department of Development and Regeneration KU Leuven Leuven Belgium

**Keywords:** acquired general lipodystrophy, anti‐PLIN1 antibodies, autoimmune hepatitis, autoimmune lipodystrophy, autoimmunity, perlipin1

To the Editor,

Generalized lipodystrophy is a rare syndrome of diffuse adipose tissue loss that may associate with metabolic and/or autoimmune comorbidities. The role of perilipin (PLIN1—a protein almost exclusively found in adipocytes) in lipodystrophy was first suggested in 2011 by the identification of deleterious genetic variants in *PLIN1* in familial forms of lipodystrophy.^1^

More recently, the presence of autoantibodies against PLIN1 has been evidenced by Corvillo et al. in patients with acquired generalized lipodystrophy (AGL).^2^ Subsequently, two cohorts of patients with AGL were studied, and autoantibodies against PLIN1 were found in 37% and 50%, respectively,^3^, ^4^ especially in patients with associated autoimmune diseases. An underlying inborn error of immunity has been reported in one patient (APECED syndrome—autoimmune polyendocrinopathy, candidiasis, ectodermal dystrophy).^4^ In vitro evidence suggests that anti‐PLIN1 autoantibodies play a pathogenic role rather than being an epiphenomenon. The proposed pathological effect of anti‐PLIN1 autoantibodies is to block the interaction between PLIN1 and αβ‐hydrolase domain containing 5 (ABHD5), causing ABDH5 to translocate to the cytosol, thereby increasing lipolysis.^3^, ^5^ While these exciting mechanistic and prevalence data are emerging, detailed clinical description of anti‐PLIN1‐positive patients are scarce in the literature. Here, we report the clinical presentation and disease course of two pediatric patients with common autoimmune diseases (type 1 diabetes, autoimmune hepatitis) who developed AGL in the presence of anti‐PLIN1 antibodies.

Patient 1 is a girl who presented at the age of 3 years with established features of generalized lipodystrophy (without prior or associated panniculitis): subcutaneous fat loss, muscular appearance for age, and phlebomegaly (Figure 1A). She also had vitiligo, acanthosis nigricans, and a cushingoid face. Personal and family history was unremarkable. Laboratory studies revealed elevated liver enzymes, hypergammaglobulinemia, negative serology tests for autoimmune hepatitis, normal C3, and slightly decreased C4 (0.13 g/L, reference range 0.16–0.38 g/L). Liver biopsy was consistent with autoimmune hepatitis (lymphocytic and plasma cells infiltration). She was treated with steroids and mycophenolate mofetil. After a first relapse, she was switched to azathioprine. Several hepatitis flares, possibly caused by poor adherence, occurred over the next 10 years and were managed with steroids and azathioprine dosage increase (Figure S1). Pronounced hypergammaglobulinemia also persisted. At 14.5 years of age, another liver biopsy harvested during hepatitis flare showed persistent hepatitis, with an elevated number of IgG4^+^ cells (Figure S1). Azathioprine was discontinued for rituximab, resulting in the current state of remission after 1.5 years on this therapy. The features of lipodystrophy have remained unchanged, without evolution throughout the disease course. Additional investigations on serum taken before receiving rituximab revealed positive anti‐PLIN1 IgG and IgM antibodies by ELISA, which were further confirmed by indirect immunofluorescence (Hospital La Paz Institute for Health Research Madrid) (Figure 1B and Figure S2).^3^ A trio whole‐exome sequencing did not reveal any pathogenic variant in genes known to cause inborn errors of immunity or inherited lipodystrophy (Table S1).

**FIGURE 1:**
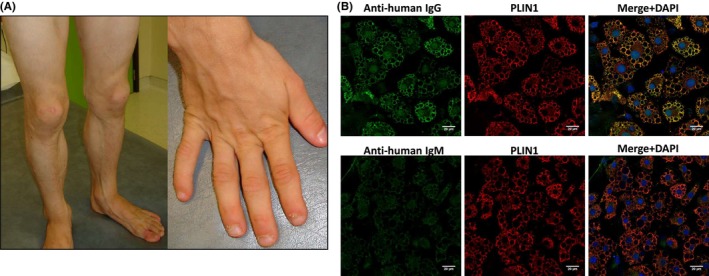
(A) Clinical photographs of patient 1 at the age of 5 years showing features of lipodystrophy: subcutaneous fat loss, muscular aspect of the lower limbs, and phlebomegaly. (B) Confocal microscopy analysis of mouse preadipocytes revealed colocalization of PLIN1 and anti‐PLIN1 IgG and IgM on the surface of lipid droplets. DNA was stained with 4′,6‐diamidino‐2‐phenylindole (DAPI, blue); IgG binding was detected with FITC‐conjugated rabbit anti‐human IgG or FITC‐conjugated rabbit anti‐human IgM (green); PLIN1 was detected with biotin‐labeled rabbit IgG followed by Texas Red‐labeled streptavidin (red). Scale bars correspond to 20 μm.

Patient 2 is a boy who was diagnosed with type 1 diabetes at the age of 1.5 years without relevant personal or family history. Three months later, his parents reported diffuse loss of subcutaneous fat with sparing of the face (Figure 2A), despite acceptable glycemic control with a subcutaneous insulin pump. There was no clinical evidence of panniculitis. Laboratory tests showed undetectable leptin concentration (<1.6 μg/L, reference range: 2.2–11.1) and C‐peptide (<0.01 nmol/L), normal C3/C4, negative autoantibodies against insulin, IA2A, GADA, and positive anti‐PLIN1 IgG antibodies by ELISA, which were further confirmed by indirect immunofluorescence (Hospital La Paz Institute for Health Research, Madrid) (Figure 2B and Figure S2).^3^ A serum sample taken and stored at diabetes diagnosis was tested and also revealed the presence of anti‐PLIN1 IgG antibodies, with similar titers (Figure 2B and Figure S2). Liver enzymes remained slightly elevated (ALT: 67–218 U/L, reference value <41) during 1 year and normalized spontaneously. Sequencing of a panel of genes involved in inherited lipodystrophy did not detect any pathogenic variant (Table S1). At the age of 3.5 years, the patient had a stable evolution with persistent generalized lipodystrophy.

**FIGURE 2:**
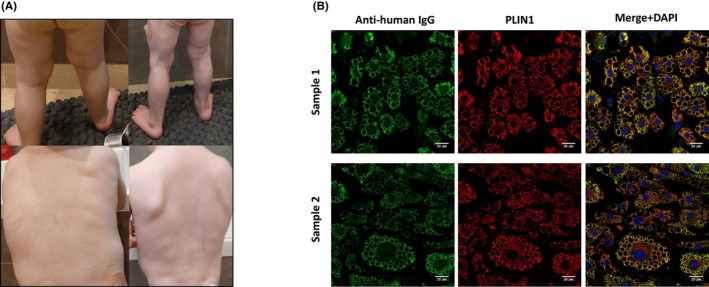
(A) Clinical photographs of patient 2, shortly after diagnosis of type 1 diabetes (left panels) and 3 months later (right panels), with severe loss of adipose tissue in the lower limbs and the back. (B) Confocal microscopy analysis of mouse preadipocytes revealed anti‐PLIN1 IgG autoantibodies in the patient in a sample 3 months before (sample 1) and after (sample 2) the onset of lipodystrophy. DNA was stained with 4′,6‐diamidino‐2‐phenylindole (DAPI, blue); IgG binding was detected with FITC‐conjugated rabbit anti‐human IgG or FITC‐conjugated rabbit anti‐human IgM (green); PLIN1 was detected with biotin‐labeled rabbit IgG followed by Texas Red‐labeled streptavidin (red). Scale bars correspond to 20 μm.

AGL with anti‐PLIN1 antibodies has been reported to be associated with one or more autoimmune diseases in 82%–85% of cases, autoimmune hepatitis being the most frequent.^3^, ^4^ Our report illustrates that AGL may be the presenting symptom or occur later in the disease course in a patient with known autoimmunity. Noteworthy, the autoantibody isotypes detected in our patients do not correlate with the clinical onset of AGL, a finding similar to previous studies.^3^ Longitudinal studies are needed to ascertain whether there is a transition from IgM to IgG during the course of the disease or, conversely, whether these two classes persistently coexist in some patients.

In addition, we provide the first evidence that anti‐PLIN1 antibodies are already present before onset of clinically apparent AGL, paving the way for screening and potential preventive interventions in presymptomatic patients with positive autoantibodies. If confirmed on additional patients, this observation might also represent an advance in the understanding of the role of anti‐PLIN1 antibodies. Indeed, some authors have raised the possibility that these autoantibodies could be a consequence of adipose tissue damage (rather than its cause) and appear secondarily to exposition of tissue‐restricted antigens.^5^ Our report would argue against the latter hypothesis, although we cannot exclude that subclinical adipose tissue loss was already present when the first sample was tested. Overall, an in vivo study of the consequences of anti‐PLIN1 antibodies remains necessary for a clear assessment of their potential pathogenic role. In conclusion, physicians caring for patients with autoimmune diseases should be aware of this rare entity, to ensure prompt diagnosis, appropriate patient information, and monitoring of comorbidities.

## AUTHOR CONTRIBUTIONS

**Clément Triaille:** Conceptualization; investigation; writing – original draft; visualization. **Fernando Corvillo:** Investigation; writing – review and editing; data curation; formal analysis. **Ann Mertens:** Writing – review and editing. **Ilse Hoffman:** Writing – review and editing; data curation; investigation. **Tania Roskams:** Investigation; writing – review and editing. **Margarita López‐Trascasa:** Investigation; writing – review and editing; data curation. **Lien De Somer:** Investigation; writing – review and editing; data curation. **Kristina Casteels:** Investigation; writing – review and editing; data curation.

## FUNDING INFORMATION

CT is partly funded by WBI World (Bourses d'excellence, Wallonie‐Bruxelles International), Fondation CHU Sainte‐Justine, and Fondation médicale Horlait‐Dapsens. FC was awarded a research fellowship by the Asociación Española de Familiares y Afectados de Lipodistrofias (AELIP).

## CONFLICT OF INTEREST STATEMENT

The authors have no competing interest to disclose in relationship with the present manuscript.

### PEER REVIEW

The peer review history for this article is available at https://www.webofscience.com/api/gateway/wos/peer‐review/10.1111/pai.70026.

## ETHICS APPROVAL

This study was approved by the ethics committee of UZ/KU Leuven (reference: S69540).

## CONSENT TO PARTICIPATE

Samples were obtained from the patients with written informed consent. Patients and/or legal representative provided written informed consent for publication of case report and clinical photographs.

## Supporting information


Figure S1.

Figure S2.

Table S1.

## Data Availability

Access to additional information can be discussed on an ad hoc basis following contact with the corresponding author.
