# Supplementary material for: Perilipin‐1 autoantibodies are a robust marker of acquired lipodystrophy and may precede clinical detection
Source: Pediatr Allergy Immunol. 2025 Jan 9;36(1):e70026. doi: 10.1111/pai.70026 (PMC11715144; doi:10.1111/pai.70026)
Supplement: Supplementary file 1 — Figure S1. [file PAI-36-e70026-s003.pdf]

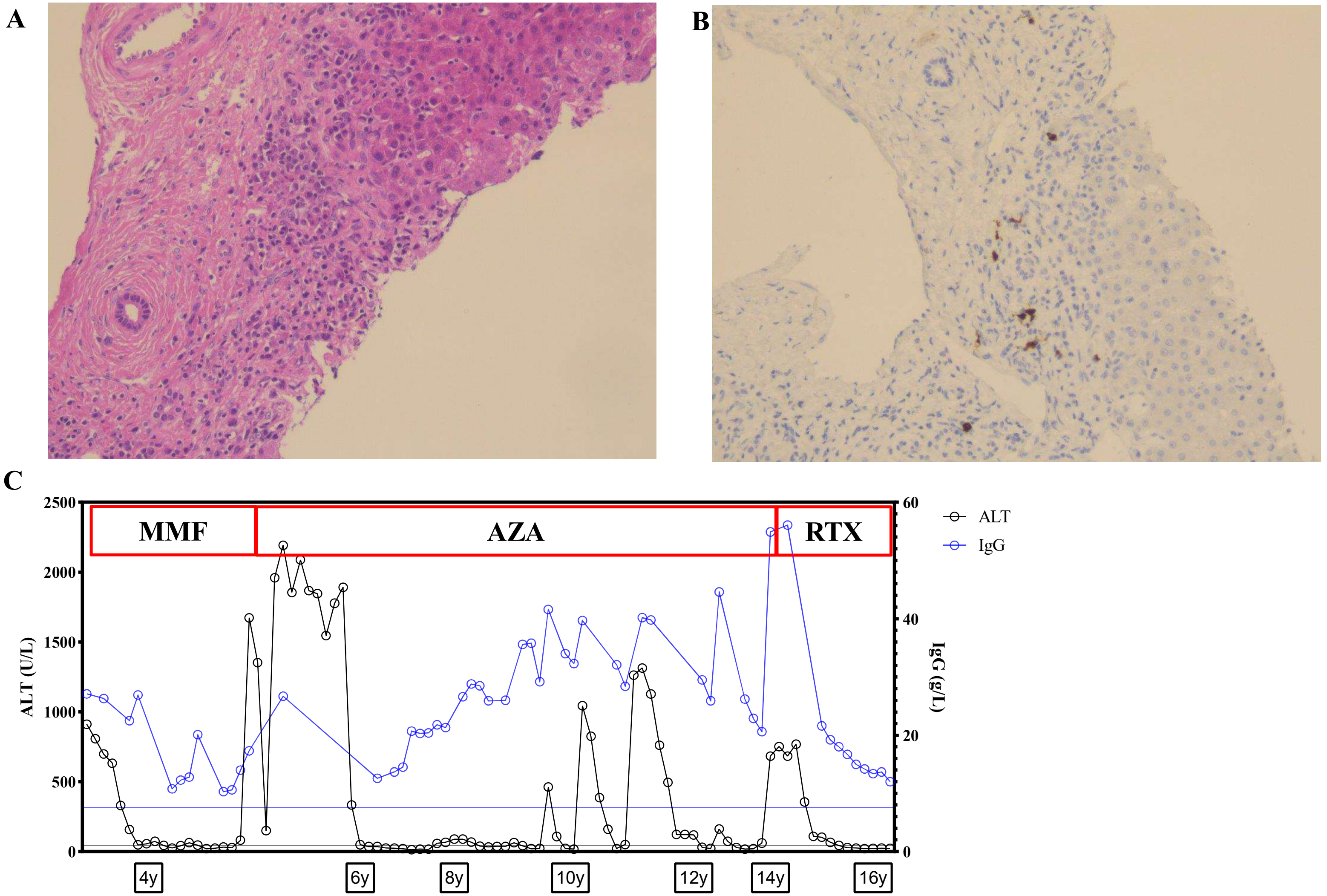

Figure S1: A) Liver biopsy image HE, x200 magnification showing a portal tract with a plasma cell rich infiltrate that infiltrates the parenchyma (interface hepatitis). B) Liver biopsy image x200 magnification positive plasma cells on IgG4 staining. C) Evolution of alanine transaminase (ALT) and immunoglobulin G (IgG) over time in patient 1. Age in years is shown below the x-axis on a non-linear scale. Steroid-sparing therapies used are shown above the graph: MMF: mycophenolate mofetil; AZA: azathioprine, RTX: rituximab.
