# Supplementary material for: Perilipin‐1 autoantibodies are a robust marker of acquired lipodystrophy and may precede clinical detection
Source: Pediatr Allergy Immunol. 2025 Jan 9;36(1):e70026. doi: 10.1111/pai.70026 (PMC11715144; doi:10.1111/pai.70026)
Supplement: Supplementary file 2 — Figure S2. [file PAI-36-e70026-s001.pdf]

A B

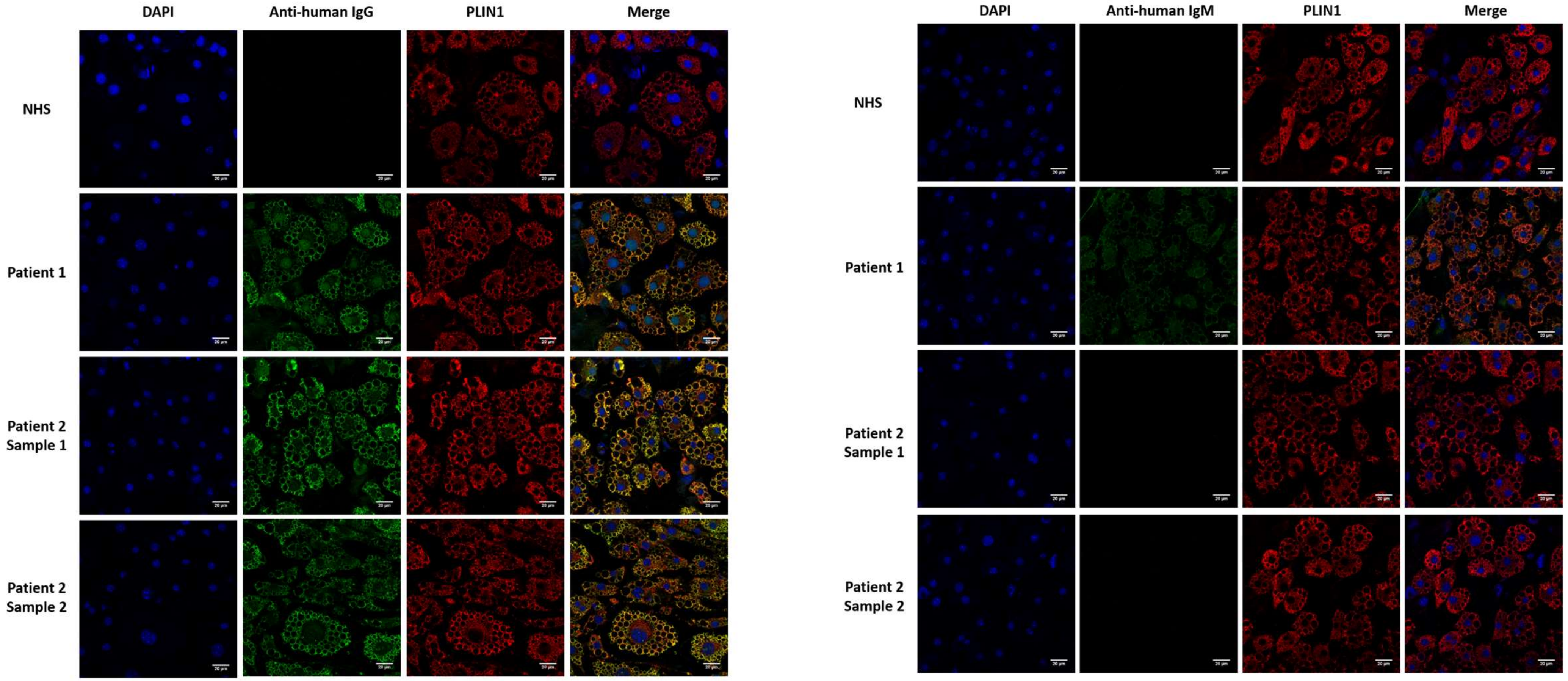

Figure S2. Immunofluorescence of anti-PLIN1 autoantibodies from the two patients with acquired generalized lipodystrophy, and a control donor (NHS) (negative for IgG and IgM anti-PLIN1 autoantibodies)**.** The 3T3-L1 line was differentiated into mouse preadipocytes, fixed and permeabilized. Under these conditions, immunofluorescence was performed. A) Confocal microscopy analysis of mouse preadipocytes revealed colocalization of PLIN1 and anti-PLIN1 IgG from patient 1 and patient 2 (in both samples) on the surface of lipid droplets. B) Confocal microscopy analysis of mouse preadipocytes revealed colocalization of PLIN1 and anti-PLIN1 IgM from patient 1 on the lipid droplet surface. However, no colocalization was observed using serum from a control donor (NHS) and patient 2 (negative for IgM anti-PLIN1 autoantibodies on ELISA). DNA was stained with 4′,6-diamidino-2-phenylindole (DAPI, blue); IgG binding was detected with FITC-conjugated rabbit anti-human IgG or FITC-conjugated rabbit anti-human IgM (green); PLIN1 was detected with biotin-labeled rabbit IgG followed by Texas Red-labeled streptavidin (red). Scale bars correspond to 20 µm.
