# Supplementary material for: Perilipin‐1 autoantibodies are a robust marker of acquired lipodystrophy and may precede clinical detection
Source: Pediatr Allergy Immunol. 2025 Jan 9;36(1):e70026. doi: 10.1111/pai.70026 (PMC11715144; doi:10.1111/pai.70026)
Supplement: Supplementary file 3 — Table S1. [file PAI-36-e70026-s002.docx]

**Supplementary Table 1**: details on genetic testing performed in the two patients

**Patient 1:**

-Gene panel of autoinflammatory disorders (Université Libre de Bruxelles, Brussels, Belgium).

Genes included in the panel: 85, see here under for details.

-Trio-whole exome sequencing performed in a research lab at UZLeuven for pathogenic variants in any potential disease-causing genes (including *AGPAT2, AKT2, BSCL2, CAV1, CIDEC, LMNA, LIPE, PLIN1, PPARG, PTRF, ZMPSTE24).*

**Patient 2**

-Gene panel of primary lipodystrophies (Universitaire Medisch Centrum, Utrecht, Netherlands).

Methods: Whole-exome sequencing followed by variants analysis (SNVs, MNVs, CNSs). Minimal coverage (15x): >99%.

Genes included in the panel*: AGPAT2, AKT2, BSCL2, CAV1, CIDEC, LMNA, LIPE, PLIN1, PPARG, PTRF, ZMPSTE24*

| **Genes included in the panel of autoinflammatory disorders (patient 1):**  *ACP5* |
| --- |
| *ADA2* |
| *ADAM17* |
| *ADAR* |
| *ADGRE2* |
| *AP1S3* |
| *CARD14* |
| *CASP1* |
| *CASP10* |
| *CDC42* |
| *CEBPE* |
| *COPA* |
| *DNASE2* |
| *DOCK8* |
| *ELF4* |
| *F12* |
| *FAS* |
| *FASLG* |
| *FBLIM1* |
| *HMOX1* |
| *IFIH1* |
| *IKBKG* |
| *IL10* |
| *IL10RA* |
| *IL10RB* |
| *IL1RN* |
| *IL36RN* |
| *LACC1* |
| *LPIN2* |
| *LSM11* |
| *LYN* |
| *MDFIC* |
| *MEFV* |
| *MVK* |
| *NCKAP1L* |
| *NCSTN* |
| *NLRC4* |
| *NLRP1* |
| *NLRP12* |
| *NLRP3* |
| *NLRP7* |
| *NOD2* |
| *OTULIN* |
| *PLCG2* |
| *POLA1* |
| *POMP* |
| *PSENEN* |
| *PSMA3* |
| *PSMB10* |
| *PSMB4* |
| *PSMB8* |
| *PSMB9* |
| *PSMG2* |
| *PSTPIP1* |
| *PTEN* |
| *PYCARD* |
| *RBCK1* |
| *RELA* |
| *RIGI* |
| *RIPK1* |
| *RNASEH2A* |
| *RNASEH2B* |
| *RNASEH2C* |
| *RNF213* |
| *RNF31* |
| *RNU7-1* |
| *SAMD9L* |
| *SAMHD1* |
| *SERPING1* |
| *SH3BP2* |
| *SHARPIN* |
| *SLC29A3* |
| *STAT2* |
| *STING1* |
| *SYK* |
| *TNFAIP3* |
| *TNFRSF11A* |
| *TNFRSF1A* |
| *TNFRSF9* |
| *TRAP1* |
| *TREX1* |
| *TRNT1* |
| *UBA1* |
| *UNC13B* |
| *USP18* |
| *WAS* |
| *WDR1* |
| *XIAP* |
